# MGA-seq: robust identification of extrachromosomal DNA and genetic variants using multiple genetic abnormality sequencing

**DOI:** 10.1186/s13059-023-03081-x

**Published:** 2023-10-30

**Authors:** Da Lin, Yanyan Zou, Xinyu Li, Jinyue Wang, Qin Xiao, Xiaochen Gao, Fei Lin, Ningyuan Zhang, Ming Jiao, Yu Guo, Zhaowei Teng, Shiyi Li, Yongchang Wei, Fuling Zhou, Rong Yin, Siheng Zhang, Lingyu Xing, Weize Xu, Xiaofeng Wu, Bing Yang, Ke Xiao, Chengchao Wu, Yingfeng Tao, Xiaoqing Yang, Jing Zhang, Sheng Hu, Shuang Dong, Xiaoyu Li, Shengwei Ye, Zhidan Hong, Yihang Pan, Yuqin Yang, Haixiang Sun, Gang Cao

**Affiliations:** 1 Precision Research Center for Refractory Diseases, Institute for Clinical Research, Shanghai General Hospital, Shanghai Jiao Tong University School of Medicine, Shanghai, China; 2 State Key Laboratory of Agricultural Microbiology, Huazhong Agricultural University, Wuhan, China; 3 College of Informatics, Huazhong Agricultural University, Wuhan, China; 4 College of Bio-Medicine and Health, Huazhong Agricultural University, Wuhan, China; 5 College of Life Science and Technology, Huazhong Agricultural University, Wuhan, China; 6 Reproductive Medical Center, Nanjing Drum Tower Hospital, The Affiliated Hospital of Nanjing University Medical School, Nanjing, China; 7 Department of Laboratory Animal Center, Shanghai General Hospital, Shanghai Jiao Tong University School of Medicine, Shanghai, China; 8 The First People’s Hospital of Yunnan Province, Affiliated Hospital of Kunming University of Science and Technology, Kunming, China; 9 Baylor College of Medicine, Houston, TX USA; 10 Department of Radiation & Medical Oncology, Zhongnan Hospital of Wuhan University, Wuhan, China; 11 Hubei Key Laboratory of Tumor Biological Behaviors, Zhongnan Hospital of Wuhan University, Wuhan, China; 12 Department of Hematology, Zhongnan Hospital of Wuhan University, Wuhan, China; 13 College of Veterinary Medicine, Huazhong Agricultural University, Wuhan, China; 14 Hospital of Huazhong Agricultural University, Wuhan, China; 15 Department of Medical Oncology, Hubei Cancer Hospital, Tongji Medical College, Huazhong University of Science and Technology, Wuhan, China; 16 Department of Gastrointestinal Surgery, Hubei Cancer Hospital, Tongji Medical College, Huazhong University of Science and Technology, Wuhan, China; 17 Dapartment of Reproductive Medicine Center, Zhongnan Hospital of Wuhan University, Wuhan, China; 18 Precision Medicine Center, Scientific Research Center, School of Medicine, The Seventh Affiliated Hospital, Sun Yat-Sen University, Shenzhen, China; 19 Shenzhen Institute of Advanced Technology, Chinese Academy of Sciences, Shenzhen, China

**Keywords:** Extrachromosomal DNA (ecDNA), Homogenously staining regions (HSRs), Structural variation (SV), Genomic abnormalities, Spatial chromatin conformation

## Abstract

Genomic abnormalities are strongly associated with cancer and infertility. In this study, we develop a simple and efficient method — multiple genetic abnormality sequencing (MGA-Seq) — to simultaneously detect structural variation, copy number variation, single-nucleotide polymorphism, homogeneously staining regions, and extrachromosomal DNA (ecDNA) from a single tube. MGA-Seq directly sequences proximity-ligated genomic fragments, yielding a dataset with concurrent genome three-dimensional and whole-genome sequencing information, enabling approximate localization of genomic structural variations and facilitating breakpoint identification. Additionally, by utilizing MGA-Seq, we map focal amplification and oncogene coamplification, thus facilitating the exploration of ecDNA’s transcriptional regulatory function.

**Supplementary Information:**

The online version contains supplementary material available at 10.1186/s13059-023-03081-x.

## Background

Genomic abnormalities, including structural variation (SV), copy number variation (CNV), focal amplification (FA) [1], single-nucleotide polymorphisms (SNPs), and INDELS (< 50 bp), are strongly associated with the development and progression of cancer [2, 3] and infertility [4, 5]. Accumulating data have demonstrated that numerous cancer cells contain extrachromosomal DNA (ecDNA), a form of FA [6, 7]. The copy number of oncogenes can be highly elevated by ecDNA-based amplification. Moreover, the chromatin architecture of ecDNA is usually highly accessible [8], which dramatically increases the expression level of oncogenes. ecDNAs can be spatially close to each other to form ecDNA hubs [9–11], which perform enhancer-like functions and increase the expression of proto-oncogenes through intermolecular interactions [9, 11, 12]. Intriguingly, in response to antitumour drug treatment, ecDNA can reintegrate back into the chromosome in another form of FA, homogenously staining regions (HSRs), via a myriad of mechanisms [13]. Increasing evidence suggests that ecDNA is associated with cancer progression and can be used as a diagnostic marker [6, 14]. However, there is no method thus far to simultaneously detect diverse types of genomic abnormalities, which greatly hampers the precise diagnosis and understanding of the molecular mechanism of cancer and genetic disease.

Based on WGS datasets, researchers developed the FA prediction software AmpliconArchitect [15] and delineated the focal amplifications and general structure of ecDNA in different types of tumours [7]. Due to the natural disadvantage of the short read length of next-generation sequencing datasets, the sensitivity of AmpliconArchitect prediction results is limited, and there is no spatial structural information of ecDNA hubs. Recently, a multiomics strategy based on second-generation sequencing, third-generation sequencing, and Hi-C has been developed to decode the spatial architecture of ecDNA hubs in detail [9]. This integrated analysis strategy can effectively decode the circular structure and spatial mobility of ecDNA. However, this strategy requires expensive multiple sequencing library construction and sequencing from the same sample, which limits its clinical application for precise diagnosis. Thus, a simple method for the simultaneous detection of different types of genomic abnormalities is crucial and highly desired for precise diagnosis and understanding the molecular mechanism of cancer and genetic disease.

To improve the detection capability of complex genomic structural variation, several new technologies have been developed [7, 16]. These technologies can be generally divided into two categories: one is based on single molecule long fragment sequencing or detection, such as Pacific Biosciences (PacBio) SMRT sequencing [17, 18], Oxford Nanopore Technologies (ONT) sequencing [16, 19, 20], and Bionano [21]; the other is based on long DNA sequence reconstruction using short read sequencing, such as strand-seq [22, 23], 10 × Genomics linked-reads [24–26]. Due to the high cost, tedious experimental steps, and large amount of initial sample, these technologies are mostly applied in scientific research, such as genome assembly [27–29], full-length transcriptome sequencing [30], and gene transcription regulation [31], but not for clinic diagnosis.

Since the invention of Hi-C technology, it has demonstrated a strong capability to detect large structural variations (SVs), including balanced and unbalanced chromosomal rearrangements, as well as copy number variations [32–34]. However, Hi-C has limitations in genome coverage, as it can only capture DNA sequence information around proximity ligation junctions. Additionally, constructing Hi-C libraries typically requires a large number of cells, despite of recent advancements aiming to improve this limitation [35, 36].

In this study, our aim was to develop an efficient and cost-effective method called multiple genetic abnormality sequencing (MGA-seq) based on standard in situ Hi-C technology [37]. MGA-Seq enables the simultaneous detection of both small mutations and large genome structural variations. To reduce the number of starting cells and simplify library construction steps, we performed all the enzymatic reactions, including restriction enzyme digestion, end-repair, and T4 DNA ligation, within a single tube. We eliminated steps involving DNA end biotin labelling, streptavidin magnetic bead enrichment, and centrifugal washing. Additionally, we directly fragmented and sequenced the proximity ligation products, to decipher both large structural variation information around proximity ligation junctions and small mutations such as single nucleotide variants (SNVs) and small indels (< 50 bp) located away from the ligation junction sites.

Using MGA-Seq, we successfully achieved the simultaneous identification of SNPs, CNVs, specific types of chromosomal translocations, and breakpoints with single-base resolution in cancer cells and blood samples from infertile patients. As MGA-Seq can locate the approximate location of genomic structural variation, it can facilitate breakpoint searching. Our study demonstrated the effective detection of extrachromosomal circular DNA (ecDNA) in tissue and cell line samples, as well as the oncogene coamplification networks. These findings may have significant implications for precise diagnosis and investigating the molecular mechanisms underlying cancer and genetic diseases.

## Results

### Overview of MGA-Seq

**Fig. 1:**
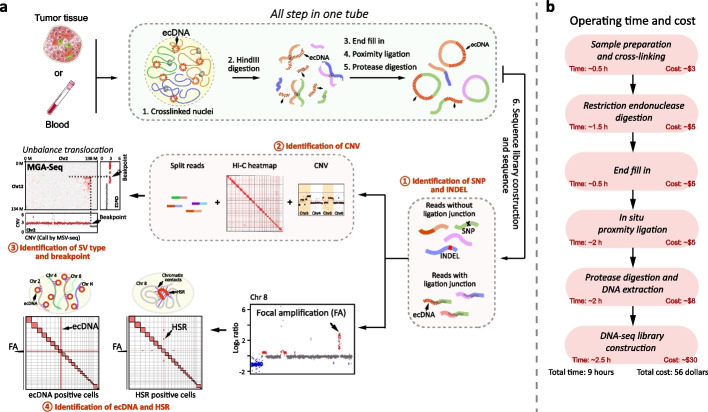
Experimental procedure, time, and cost of multiple genetic abnormalities sequencing (MGA-Seq). **a** Flowchart of MGA-Seq. Nuclei were cross-linked with 0.5% formaldehyde and then digested with HindIII. 5′ DNA overhangs of digested chromatin fragments were filled in by DNA polymerase and then proximity ligated by T4 DNA ligase. The proximity ligation products were fragmented and then subjected to high-throughput sequencing library construction. After sequencing, all the reads were used to generate a chromatin contact matrix for genome structural variation calling. In the sequencing library, the reads without ligation junction “AAGCTAGCTT” were used for the detection of CNV, SNP, small indels (< 50 bp), region of focal amplification, and genome breakpoints. By combining all information, the types and breakpoints of structural variation can be decoded. Notably, MGA-Seq can distinguish ecDNA and HSR, predict the structure of simple focal amplification regions, and construct the interaction network of focal amplificated genes. **b** The main steps, time, and cost of MGA-Seq

To maintain the spatial architecture of the genome, the nuclei are first fixed by formaldehyde in multiple genetic abnormality sequencing (MGA-Seq). The genome is digested in situ by restriction endonuclease followed by 5′ DNA overhang fill-in by DNA polymerase I. Next, the spatially adjacent chromatin fragments are proximity ligated using T4 DNA ligase and then fragmented into a high-throughput sequencing library (Fig. 1a). This library contains two kinds of sequencing reads. The reads without proximity ligation junctions were used to detect SNPs, CNVs, small inserts and deletions (< 50 bp), focal amplification (FA), and genomic breakpoints (Fig. 1a; Additional file 1: Fig. S1). As the reads with proximity ligation junctions contain spatially adjacent chromosome fragment contact information of the genome, they can be used to decode chromosome structure. Thus, the integrated analysis of all the sequencing reads can identify large chromosome structural variations, such as balanced and unbalanced translocations, extrachromosomal DNA (ecDNA), and intrachromosomal homogenously staining regions (HSRs). Notably, all MGA-Seq steps are carried out in the same tube and do not require buffer replacement, which takes only 9 h and costs just 56 dollars (Fig. 1b).

### Identification of SNPs and indels by MGA-Seq

**Fig. 2:**
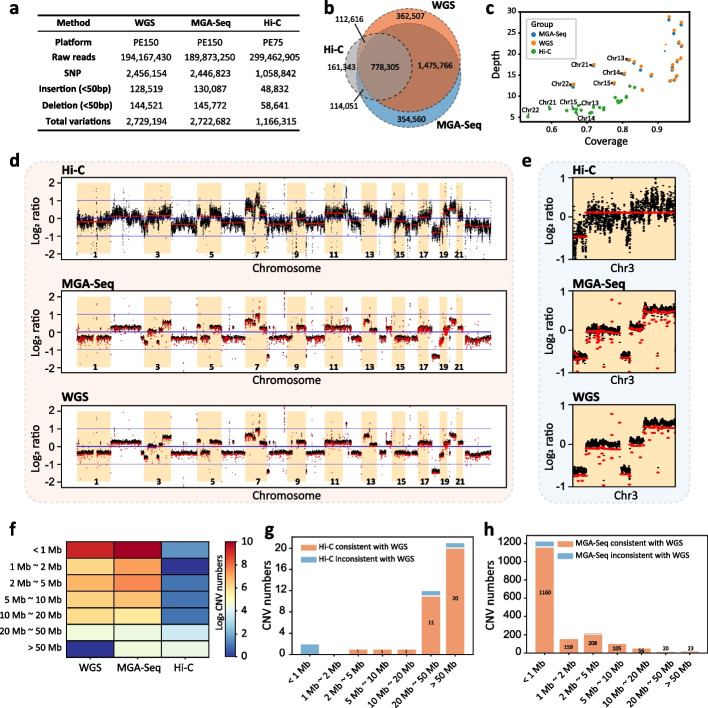
Detection of SNPs, indels, and CNVs in the SW480 cell line using MGA-Seq.** a** Comparison of the numbers of SNPs and indels (< 50 bp, include insertions and deletions) identified by WGS, MGA-Seq, and Hi-C. **b** Overlap of the SNPs and indels between MGA-Seq, WGS, and Hi-C. **c** Scatter plot of sequencing depth and coverage for each chromosome. Blue points represent MGA-Seq, yellow points represent WGS, and green points represent Hi-C. *X*-axis represents coverage, and *Y*-axis represents sequencing depth. The five acrocentric chromosomes (13, 14, 15, 21, and 22) possess extensive repetitive regions within their short arms, leading to relatively lower coverage in chromosome mapping, as indicated in the figure. **d** Comparison of log_2_ copy ratios calculated by BIC-SEQ2. The CNV segmentation is plotted in red with log_2_ copy ratio, and the black dot represents the log_2_ copy ratio per bin. **e** Comparison of the CNVs on chromosome 3 identified by Hi-C, MGA-Seq, and WGS. **f** Statistics of the number and size distribution of CNVs identified by Hi-C, MGA-Seq, and WGS. **g** Consistency of the CNV segments (categorized by size) detected by Hi-C and WGS. Overall, Hi-C cannot detect CNV with length less than 20 Mb. **h** Consistency of the CNV segments detected by MGA-Seq and WGS. The number and size distribution of CNV segments detected by MGA-Seq and WGS are highly consistent, especially for micro-CNVs (< 1 Mb)

To evaluate the SNP and indel detection capability, we performed MGA-Seq on the colorectal cancer cell line SW480 as described in Fig. 1a. After sequencing, we obtained 194,167,430 read pairs, of which 2,982,113 (1.5%) read pairs contained “AAGCTAGCTT” ligation junction sequences. To avoid false positives caused by ligation junctions, we filtered out this part of the reads for SNP and indel detection (see the “Methods” section) and analysed the remaining reads by the Genome Analysis Toolkit (GATK). To evaluate the SNP and indel variation calling efficacy, we used the SW480 cell line to generate standard WGS datasets and downloaded the SW480 in situ Hi-C datasets [38] for comparison with the same parameters (see the “Methods” section). As shown in Fig. 2a, MGA-Seq identified 2,722,682 variants, including 2,446,823 SNPs, 130,087 insertions, and 145,772 deletions. A total of 82.8% of these variants were consistent with WGS (Fig. 2b). Hi-C found only 1,166,315 variants, which is much lower than that identified by MGA-Seq and WGS (Fig. 2a). Furthermore, the sequencing coverage and depth of MGA-Seq were also much higher than those of Hi-C (Fig. 2c and Additional file 1: Fig. S2a and b).

### Detection of chromosome copy number variation by MGA-Seq

To test the CNV detection capability of MGA-Seq, we plotted the log_2_ ratio of average read depths in 50-Kb bins across the genome, as shown in Fig. 2d. Our data showed that the genome coverage and uniformity of MGA-Seq are highly consistent with the gold standard WGS datasets and much higher than those of the Hi-C datasets. After zooming in on chromosome 3, we observed that Hi-C roughly divided chromosome 3 into two CNV intervals, whereas MGA-Seq accurately identified all the small copy number variation across the whole chromosome (Fig. 2e). Next, we systematically analysed the size and number of CNVs identified by these three methods (Fig. 2f–h) and found that it was extremely difficult to detect CNVs less than 10 Mb by Hi-C (Fig. 2f, g). In this scenario, the CNV detection capability of MGA-Seq is much better than that of Hi-C, especially for micro-CNVs (< 1 Mb), which is highly consistent with WGS (Fig. 2f, h).

### Identification of chromosomal translocations and breakpoints by MGA-Seq with single base-pair resolution

**Fig. 3:**
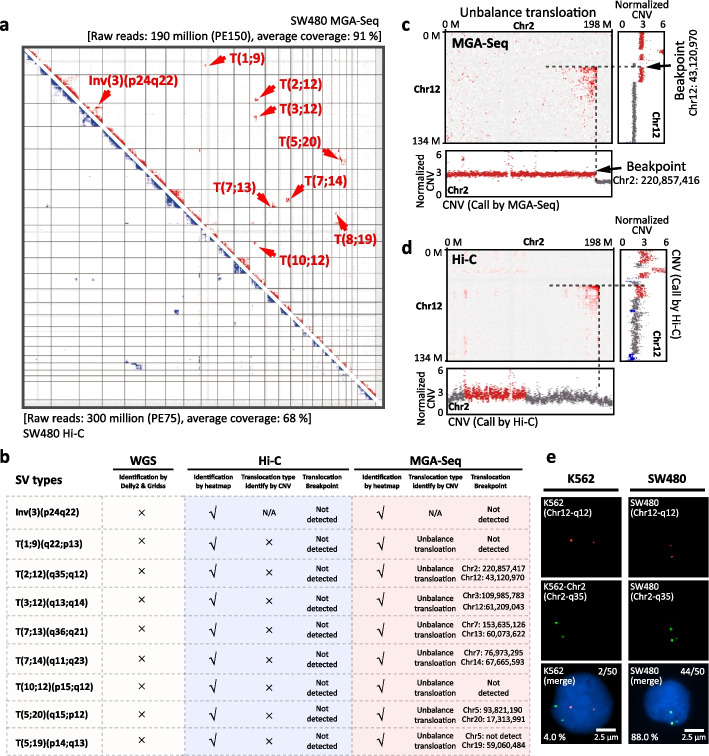
Identification of translocation types and breakpoints in SW480 at single base-pair resolution by MGA-Seq. **a** Identification of translocation in the SW480 cell line by genomic contact matrix constructed with MGA-Seq and Hi-C datasets. The detected structural variations are indicated by arrows. **b** Translocation types and breakpoint information identified by MGA-Seq. **c** Application of integrated chromatin contact matrix, CNVs, and split reads analysis to identify translocation types and breakpoints between chr 2 and chr 12 at single base-pair resolution using MGA-Seq datasets. **d** Identification of translocation types and breakpoints between chr 2 and chr 12 using Hi-C datasets. **e** Validation of the T(2;12)(q35;q12) translocation in SW480 cells by DNA FISH. FISH probes for 12q12 and 2q35 were directly labelled with Alexa Fluor 555 (red) and Alexa Fluor 488 (green), respectively. K562 cells without the T(2;12)(q35;q12) translocation were used as a control. 4.0% (2/50) 12q12 loci (red) were colocated with 2q35 loci (green) in K562 cells and 88.0% (44/50) 12q12 loci (red) were colocated with 2q35 loci (green) in SW480 cells. Each experiment was replicated three times

By using SW480 MGA-Seq sequencing datasets, we obtained the genome-wide chromosome contact matrix. As shown in Fig. 3a, b, we identified 8 translocations and 1 inversion. Although MGA-Seq only used 190 million raw reads, the structural variants detected by MGA-Seq were completely consistent with in situ Hi-C with 300 million raw reads (Fig. 3a). To further identify the chromosomal translocation types and breakpoints of these translocations, we combined chromosome contact matrix, CNV, and split read information from MGA-Seq datasets and performed integrated analysis. Taking T(2;12)(q35;q12) as an example, from the CNV data, we observed that the copy number of chromosome 12 was increased, whereas the copy number of chromosome 2 was decreased downstream of the chromosome breakpoint (Fig. 3c), suggesting that unbalanced translocation occurred between chromosomes 2 and 12.

Based on the split reads information in MGA-Seq, we further identified that the translocation breakpoint is located at chr2: 220,857,416 and chr12: 43,120,970 (Fig. 3b, c). In contrast, due to the low genome coverage and depth of Hi-C, it is not feasible to precisely determine the type and breakpoint of translocation (Fig. 3b, d). In this scenario, MGA-Seq identified that all 8 chromosomal translocations in the SW480 cell line were unbalanced translocations. Notably, we were able to pinpoint the breakpoints of 6 out of 8 translocation sites at single-base resolution (75.0%). We also used WGS data with the same sequencing depth as MGA-Seq to identify the translocations. As there is no chromosome interaction information in this dataset, none of the chromosomal translocations were found (Fig. 3b). Moreover, we verified the T(2;12)(q35;q12) translocation by two-colour DNA fluorescence in situ hybridization (FISH). As shown in Fig. 3e, chromosomes 2 and 12 were indeed fused together in SW480 cells, supporting the integrity of MGA-Seq.

Furthermore, to test the chromosomal translocation detection capability of MGA-Seq in clinical samples, we collected peripheral blood from two infertile patients with known translocation sites and constructed an MGA-Seq library. By combining the chromosome interaction matrix and CNV data, we detected a T(10;22)(p12;q13) translocation in sample 1 (Additional file 1: Fig. S3a) and a T(9;11)(q21;p14) translocation in sample 2 (Additional file 1: Fig. S3b), which are consistent with the known translocation sites identified by karyotyping. In addition, based on the split reads, we pinpointed the precise location of the breakpoints with single base-pair resolution (Additional file 1: Fig. S3a, b). Next, we analysed the CNV information of these two samples based on the MGA-Seq data to determine the translocation type. Our data showed that there are no chromosome copy number changes around the translocation breakpoint, meaning that both infertile patients carry balanced translocations. Together, these data demonstrated that MGA-Seq can detect specific chromosomal translocation types and the corresponding breakpoint with high efficacy and low cost.

### Detection of ecDNA by MGA-Seq

**Fig. 4:**
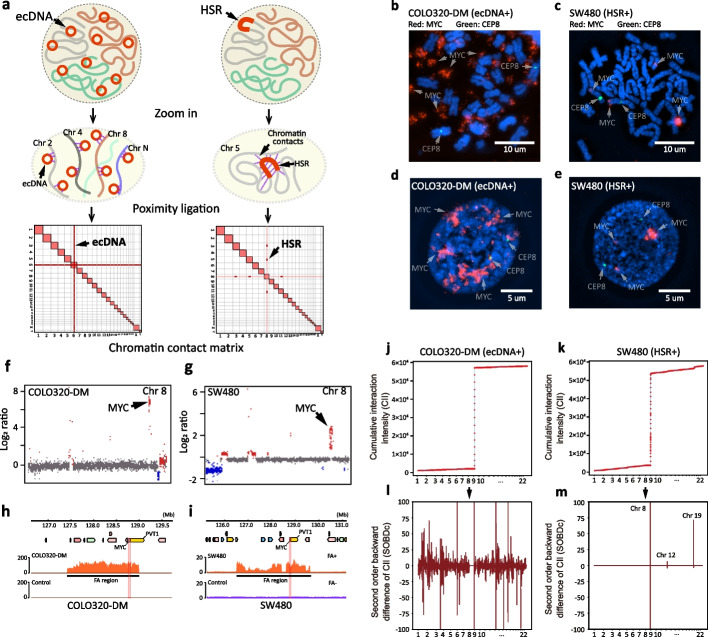
Identification of ecDNA by MGA-Seq.** a** Putative diagram of inter-chromosomal interaction pattern differences between ecDNA and HSR positive cell line. **b-e** Validation of *MYC* amplification in COLO320-DM and SW480 cell lines by DNA FISH. The red signal represents *MYC* and the green signal represents the centromere of chr 8. **f**, **g** Copy number variation analysis of chr 8 in COLO320-DM and SW480 cell lines. Gains and losses of copy numbers are shown in red and blue, respectively. **h**, **i** Location of the *MYC* amplification region in COLO320-DM and SW480 cell lines. The *y*-axis represents the reads counts that were normalized based on the total number of reads mapped per sample. **j**, **k** Cumulative interaction intensity curve of focal amplification region from COLO320-DM (chr8:127,300,000–128,900,000) and SW480 (chr8: 126,493,801–129,610,570) respectively. The *x*-axis represents the genome position, 100 kb bin size. The *y*-axis represents the accumulation of interaction intensity. **l**, **m** Plotted the second-order backward difference (SOBD) values across the genome for the focal amplification region of COLO320-DM (chr8:127,300,000–128,900,000) and SW480 (chr8: 126,493,801–129,610,570) using a bin size of 100 kb

Due to the high mobility and dramatic amplification amount of ecDNA, from bulk cells sequence results we speculated that ecDNA can contact with each chromosome with a significantly higher interaction frequency than the normal interchromosome interaction (Fig. 4a). To prove this hypothesis, we selected the ecDNA-positive cell line COLO320-DM [8] and the HSR-positive cell line SW480 [39] for MGA-Seq analysis. First, *MYC* amplifications in the form of ecDNA in COLO320-DM cells and in the form of HSR in SW480 cells were confirmed by DNA FISH (Fig. 4b, c). In comparison to HSR-positive SW480 cells, ecDNA-positive COLO320-DM cells showed *MYC* amplification throughout the nucleus (Fig. 4d, e). Furthermore, CNV analysis based on the MGA-Seq dataset accurately located the *MYC* amplification regions in these two cell lines (Fig. 4f–i).

Next, we constructed the chromatin interaction matrix using MGA-Seq data. Since the amplified ecDNAs were distributed throughout the nucleus (Fig. 4d), the ecDNA fragments were ligated to all the chromatin fragments upon proximity ligation and thus presented a strip-like structure in the whole chromatin contact matrix (Additional file 1: Fig. S4a). In contrast, as HSR is amplified on specific chromosomal regions (Fig. 4c, e), it only shows strong interchromosomal interactions on certain chromosomes (Additional file 1: Fig. S4b), which is consistent with our hypothesis (Fig. 4a). In addition, we observed the same interchromosomal interaction pattern in ecDNA-positive cell lines TR14 and SNU16 [8, 9] (Additional file 1: Fig. S4c, d). From the interchromosomal interaction matrix of SW480, we found that the *MYC* focal amplification region has a strong interaction with 19q13.3, indicating that *MYC* is likely to be amplified on chr19 (Additional file 1: Fig. S4e, f). This finding is consistent with a previous report [39].

Since the judgement dependent on the naked eye is subjective and differs among individuals, we performed genome-wide interaction fluctuation analysis (GWIFA) on the focal amplification regions (Fig. 4j–m) for a more objective identification of ecDNA (see the “Methods” section). First, we divided the genome into fixed-size bins and calculated the interaction intensity between the amplified region and each bin. The cumulative interaction intensity curve was then plotted as shown in Fig. 4j, k. Next, second-order backwards difference (SOBD) analysis was applied to evaluate the fluctuation of the cumulative interaction intensity curve (Fig. 4l, m). As HSR is amplified on the specific chromosome, the value of SOBD fluctuates dramatically at specific genomic locations (Fig. 4m). However, ecDNA has strong interactions with distinct strengths across the whole genome. Thus, the value of SOBD fluctuates greatly throughout the whole genome (Fig. 4l; Additional file 1: Fig. S4g, h).

### Delineation of the architecture of focal amplification in K562 cells

**Fig. 5:**
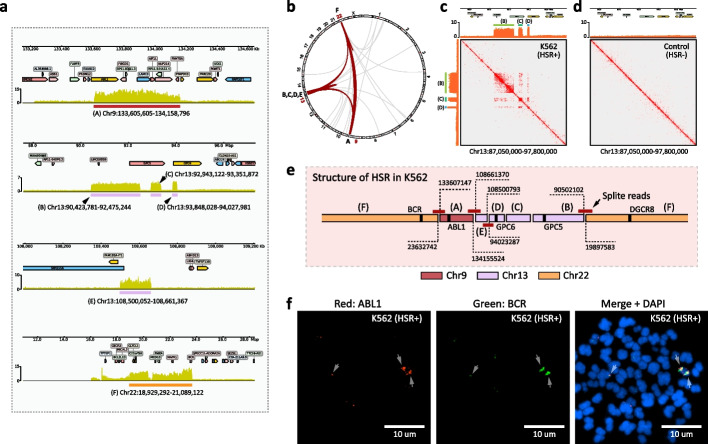
Deciphering the spatial structure of the homogenously staining region (HSR) in the K562 cell line. **a** Location of the amplification region on chr 9, 13, and 22. The *y*-axis represents the reads count that was normalized based on the total number of reads mapped per sample. **b** Circos plots of the chromatin interactions mediated by amplification regions across all 23 chromosomes in K562 cell lines. The interactions between chromosomes 9, 13, and 22 are marked with red lines. **c,d** Comparison of chromatin contact matrix of amplification region (Chr13:90,423,781–92,475,244, Chr13:92,943,122–93,351,872, and Chr13:93,848,028–94,027,981) between K562 cell line and healthy human peripheral blood cells (control). **e** Assembling the amplified regions from “A” to “F” with split reads. The breakpoint of the amplification regions is marked in the figure. **f** Metaphase analysis and DNA FISH to validate the location of the *ABL1* amplification region and the *BCR* amplification region in the K562 cell line. FISH probes for the *ABL1* amplification region and the *BCR* amplification region were directly labelled with Alexa Fluor 555 (red) and Alexa Fluor 488 (green), respectively

Our MGA-Seq analysis of K562 cells identified an abnormal increase in chromosome copy number on specific regions on chromosomes 9, 13, and 22 (Additional file 1: Fig. S5a). After zooming in on the abnormally amplified regions, we identified six precisely amplified subregions, one on chromosome 9, four on chromosome 13, and one on chromosome 22, which were named “A” to “F,” respectively (Fig. 5a). Based on genome-wide interaction fluctuation analysis (GWIFA), we found that these regions were amplified in K562 cells in the form of HSR rather than ecDNA (Additional file 1: Fig. S5b). Notably, we observed strong interactions between these amplified regions, suggesting that these regions are spatially close together, which likely originate from the same HSR (Fig. 5b). Taking the “B,” “C,” and “D” amplified regions of chromosome 13 as examples, these three regions are in high contact with each other and form a high-density topologically associating domain (TAD)-like structure [40] (Fig. 5c). Such abnormal genome amplification and TAD-like structures were absent in healthy human peripheral blood cells (Fig. 5d).

Since the MGA-Seq dataset contains whole-genome sequencing information, we extracted the split reads located at the boundaries of these six amplified regions (Additional file 2: Table S1) and assembled the structure of HSR. In the K562 cell line, *ABL1* in the “A” amplification region, *GPC5* in the “B” amplification region, *GPC6* in the “D” amplification region, and *DGCR8* and *BCR* in the “F” amplification region were spliced to form a repeating HSR (Fig. 5e; Additional file 1: Fig. S6). To validate the HSR structure predicted by MGA-Seq, we compared our predicted results with published K562 third-generation sequencing data [41]. Our analysis showed that *ABL1* in chromosome 9, *GPC5* and *GPC6* in chromosome 13, and *DGCR8* and *BCR* in chromosome 22 indeed come from the same scaffold, which is highly consistent with our results. Finally, we applied DNA FISH to verify the spatial location of the *ABL1* amplification region on Chr9 and the *BCR* amplification region on chromosome 22. As shown in Fig. 5f, *ABL1* and *BCR* indeed come from the same HSR.

### Identification of focal amplification in tumour tissue

**Fig. 6:**
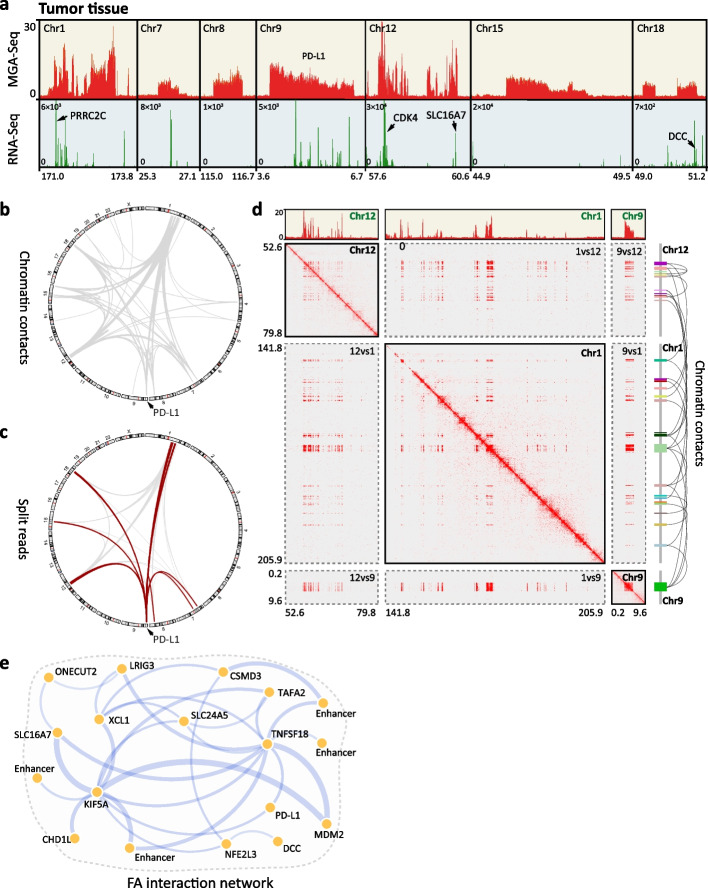
Heterogeneity of focal amplification in renal cancer tissue. **a** Sequencing reads depth and RNA expression level in typical focal amplification regions of a renal cancer tissue sample. The *y*-axis represents the reads count that was normalized based on the total number of reads mapped per sample. **b** Circos plots of the chromatin interactions mediated by focal amplification regions across all 23 chromosomes in renal cancer tissue. **c** Circos plots of the split reads mediated by focal amplification regions across all 23 chromosomes. The split reads aligned to the PD-L1 amplified region are marked with red lines. **d** Chromatin contact matrix between the amplified regions of chr1, chr9, and chr12, and sequencing reads depth within these amplified regions. **e** Coamplification network of amplified oncogenes in the renal cancer tissue sample. Different amplified oncogenes are assembled by split reads. The thickness of the line indicates the chromatin contact strength

Next, we applied MGA-Seq to tumour samples and detected 40 focal amplification regions in one renal cancer tissue (Additional file 1: Fig. S7; Additional file 3: Table S2). The length distribution of these regions varies from 4.2 Kb to 2.53 Mb (Additional file 3: Table S2). These amplified regions contain a large number of immune genes, oncogenes, and enhancers, such as *CDK4*, *SLC16A7*, *PRRC2C*, and *HMGA2* (Fig. 6a; Additional file 3: Table S2). In addition, the RNA transcription level of these genes within the amplified region was significantly higher than that of the normal kidney tissue control (Fig. 6a; Additional file 1: Fig. S8a). Through MGA-Seq chromatin contact matrix and GWIFA (Additional file 1: Fig. S8b, c), we identified that these FA regions are amplified in the form of HSR. Of note, these amplified regions are not independent but contact each other at the spatial level (Fig. 6b).

FA in tumour tissue is highly heterogeneous compared to single-cell-derived cell lines. Taking the *PD-L1* amplification region on chromosome 9 in this tumour as an example, this region can be spliced with multiple FA regions, as indicated by the split reads in the MGA-Seq dataset, suggesting that multiple types of HSR coexist in this heterogeneous tumour tissue (Fig. 6c). To verify this result, we performed single-molecule nanopore sequencing on the same tumour sample, which revealed highly consistent inter- and intrachromosomal structural variation as with the MGA-Seq dataset (Additional file 1: Fig. S8d). The inter- and intrachromosomal interaction analysis of chr1, chr9, and chr12 amplification regions based on the MGA-Seq chromatin contact matrix (Fig. 6d) identified highly complicated and heterogeneous spatial architectures of these FA regions. For example, chr1 and chr12 show an “uneven amplification” pattern, meaning that in a certain chromosome interval, only some regions were amplified, such as the regions containing proto-oncogenes, immune genes, and some regulatory elements (Fig. 6a and d). These genes and regulatory elements are spliced together and eventually form a variety of HSRs. Based on the chromatin contact information and split reads, we constructed a coamplification network of these amplified oncogenes (Fig. 6e). From this network, we can recognize which oncogenes or transcriptional regulatory elements tend to splice together to form HSR and the spatial interaction strength of these amplified regions. Taking *TNFSF18* for instance, this region was spliced and co-amplified with the other 11 amplified regions and has the strongest interaction strength with the *MDM2* amplified region.

## Discussion

ecDNA is prevalent in at least 30 different cancer types, is closely associated with cancer progression [12, 42], and might be used as a potential prognostic marker. However, there is still a lack of an unbiased and efficient detection method in clinical practice. While AmpliconArchitect can be used for ecDNA prediction [15], the identification of ecDNA based on the WGS dataset is generally limited in sensitivity. For instance, in the cell line K562 in this study, due to the head-to-tail tandem duplication HSR structure (Fig. 5e), a large number of split reads also presented a circle junction-like structure. Circle-Seq can effectively analyse the structure of circular DNAs [43, 44]. However, the DNA extraction process of this method can easily destroy the circular structure of ecDNA. Moreover, Circle-Seq is based on rolling-circle DNA amplification, it preferentially amplifies smaller circular DNAs, resulting in biased amplification results. Here, we demonstrated that MGA-Seq can unbiasedly detect the presence of ecDNA in both cell lines and clinical samples. Importantly, we proposed an ecDNA detection algorithm, GWIFA. Of note, MGA-Seq can reveal trans interactions between ecDNA and the genome, which could facilitate the exploration of the regulatory role of ecDNAs.

Chromosomal translocations can be divided into unbalanced and balanced translocations. Unbalanced translocation usually occurs with an altered chromosomal copy number at the breakpoint (gain or loss of genetic material), resulting in abnormal gene expression. A large number of unbalanced translocations have been found in cancer cells [45, 46], especially in blood tumour genomes [47, 48]. Balanced translocations do not have any genetic material changes. These translocation carriers usually have normal phenotypes and intelligence but can produce various unbalanced rearranged gametes during germ cell meiosis, resulting in infertility, abortion, stillbirth, and multiple malformations [5, 49, 50]. Thus far, it is still challenging to precisely identify the specific translocation types by a simple and cost-effective method. As MGA-Seq contains CNV and chromatin contact information, it can guide translocation breakpoint searching and facilitate to identifying translocation types and breakpoints. Here, we revealed the translocation types and breakpoints of infertile couples by MGA-Seq. With this important information, high-quality blastocysts can be quickly screened by PCR before blastocyst transfer during in vitro fertilization, which greatly reduces the cost and time of traditional whole genome sequencing for each blastocyst.

Notably, the co-existence of ecDNA and HSR in certain tumour samples poses a challenge for the current MGA-Seq method as it cannot be effectively distinguished in this scenario. Therefore, it is imperative to further improve the algorithm and experiment method to accurately differentiate these complex types of focal amplifications. Additionally, the relatively low sequencing depth of chromatin interaction information in MGA-Seq limits its ability to detect small-scale intra-chromosomal structural variations. Hence, MGA-Seq is better suited for detecting structural variations between chromosomes or large-scale variations within a single chromosome.

Together, we have developed a simple, cost-effective, and robust MGA-Seq method that enables the simultaneous detection of SNPs, CNVs, SVs, and the spatial architecture of FA by a single-tube assay. The consistency of MGA-Seq results in two independent replicate experiments, shown in Additional file 1: Fig. S9a–d, further demonstrates the reliability and robustness of this method. We have successfully utilized this technique to identify both small SNPs/INDELs and large genomic structural variations in clinical sample and cell lines, decoded the spatial architecture of focal amplification in K562 cell lines, and constructed co-amplification networks of the amplified oncogenes in clinical renal cancer tissues. Our data revealed that focal amplification is highly diverse in tumour tissues compared to single-cell-derived cancer cell lines. In the future, it would be important to develop single-cell MGA-Seq for diverse ecDNA detection in single cells or highly heterogeneous cancer cells. With its multifunctional and cost-effective advantages, we expect MGA-Seq to be extensively applied for the diagnosis of cancer and infertility, and it may greatly facilitate the investigation of the genomic mechanisms for genetic diseases.

## Conclusions

In this study, we introduced MGA-Seq, a simple and cost-effective method for simultaneously detecting SNPs, CNVs, SVs, ecDNA, and HSRs in a single tube. This method has been successfully applied in both cancer cell lines and clinical tumour samples, revealing substantial heterogeneity in focal amplification in tumour tissue. MGA-Seq can unbiasedly detect the presence of ecDNA in both cell lines and clinical samples. Importantly, we proposed an ecDNA detection algorithm. As MGA-Seq contains CNV and chromatin contact information, it can facilitate translocation breakpoint searching and accurately identify translocation types and breakpoints. MGA-Seq can reveal trans interactions between ecDNA and the genome, decode the ecDNA and HSR spatial structure, and construct coamplification networks of oncogenes in cancer tissue, which could facilitate the exploration of gene regulation inside ecDNA.

## Methods

### MGA-Seq library construction

#### Preparation of cell suspension

For tumour tissue, 0.5-cm^3^ tissue blocks were used and minced through a 40 μm strainer to obtain single-cell suspension. For blood samples, we directly took 1 ml of anticoagulated whole blood and centrifuged at 1500 g/min for 10 min to collect blood cells.

#### Nuclei preparation

Cells were cross-linked with 0.5% formaldehyde (Sigma) for 10 min. The cross-linking reaction was terminated by glycine at a final concentration of 200 mM and lysed in lysis buffer (PBS contain 0.2% SDS) at room temperature for 5 min. After incubation, the nuclei were pelleted by centrifugation at 2000 g/min for 5 min. The nuclei were transferred to 1.5 ml tubes and washed twice with PBS.

#### In situ digestion

For in situ restriction enzyme digestion, 140 μl of ddH_2_O, 20 μl of 10% Triton X-100, 20 μl of 10 × NEBuffer 2.1, and 20 μl of HindIII (NEB, 20 units/μl) were added to the nuclei pellet and digested for 1.5 h at 37 °C in thermomixer (Eppendorf) with rotation at 1000 r.p.m.

#### End filling-in

Add 5 μl of dNTP mix (10 mM each) and 5 μl of DNA polymerase I Klenow fragment (NEB, M0210) to the reaction system, place the sample in thermomixer with rotation at 37 °C at 1000 r.p.m for 30 min.

#### In situ* proximity ligation*

Add 27.5 μl of H_2_O, 3 μl of ATP (adenosine-triphosphate, 10 mM), and 10 μl of T4 DNA ligase (Thermo, EL0011) to the reaction system, and place the tube on the rotating mixers for 2 h at room temperature with rotation at 20 r.p.m.

#### Reversal of cross-linking and DNA purification

Add 20 μl of proteinase K (20 μg/ml) to the proximity ligation system, and then incubate at 60 °C for 2 h. After digestion, the DNA was directly extracted using PCR Purification Kits (Zymo, D4013).

#### Sequencing library construction

DNA sequencing libraries were prepared using the VAHTS Universal Plus DNA Library Prep Kit (NDM627) according to the manufacturer’s protocol.

### Metaphase analysis and DNA fluorescence in situ hybridization (FISH) assay

SW480 and COLO320-DM cell lines were treated with colchicine at final concentration 8 μg/ml for 24 h. After cultivation, cells were collected by centrifugation at 1000 g/min for 10 min. Next, 10 ml of hypotonic KCl solution (0.075 M) was added to the cell pellet to resuspend the cells. After 30 min incubation at 37 °C, 2 ml of fixative (3:1 methanol:glacial acetic acid) was added to the cell suspension. The cell pellet was re-collected by centrifugation at 1000 g/min for 10 min and then resuspended in 5 ml of fixative (3:1 methanol:glacial acetic acid). After 5 min incubation, the cell pellet was re-collected by centrifugation at 1000 g/min for 10 min and resuspended in 1 ml of fixative. After fixation, 10 μl of the suspension was dropped on the glass slide and incubated in the prewarmed 2 × SSC at 60 ℃ for 30 min. The cells were dehydrated sequentially in 70%, 85%, and 100% ethanol solution. After ethanol dehydration, the cells were heated on a hot plate at 82 °C for 10 min in 80% formamide (Sigma) and 2 × SSC for DNA denaturation. Next, cells were incubated for 12 h in a hybridization solution with 2 μM DNA probes (MYC and CEP8, Spatial FISH Co. Ltd.) in the presence of 50% formamide, 8% dextran sulphate sodium salt (Sigma), and 2 × SSC. After hybridization, the cells were washed three times with 20% formamide and 3 times with 2 × SSC. Finally, the slides were stained with DAPI (Life Technologies) and observed under a super-resolution microscope (Nikon, N-SIM).

### RNA-Seq library preparation

RNA was extracted using the RNAiso Plus (Takara, 9109) according to the manufacturer’s protocol. Sequencing libraries were prepared using the VAHTS Stranded mRNA-Seq Library Prep Kit (Vazyme, NR602-02) according to the manufacturer’s protocol.

### Identification of SNP, indel, split reads, and CNV using MGA-Seq datasets

#### Pre-analysis

FastQC [51] (version: 0.11.5) was used to assess the quality of raw reads. FASTP [52] (version: 0.23.2) was used to filter out the low-quality bases and adapter sequences. The clean read pairs which contained proximity ligation junction sequences “AAGCTAGCTT” were filtered out by the Linux command line utility “grep”. The remaining reads were used for SNPs, indels, split reads, and CNV calling.

#### SNP and indel calling

The remaining reads were aligned to the reference genome (hg19) and generated a BAM file using BWA-MEM [53] (version 0.7.17). The BAM file was sorted by SAMtools [54] (version 1.15.1) and deduplicated by Sambamba [55] (version 0.6.6). Next, we used BaseRecalibrator (GATK [56], version 4.2.2) to calibrate the base quality scores, and HaplotypeCaller (GATK, version 4.2.2) to detect SNPs and indels.

#### Split reads calling

The deduplicated BAM file generated in the SNP and indel calling step were used to identify split reads. The split alignment reads were extracted by SAMtools (version 1.15.1) with the command line “samtools view test_deduplicated.bam | grep SA > test_split_reads.txt”.

#### CNV calling

BIC-seq2 (version 0.7.2) [57] was used to derive CNV segments from reads coverage data. For more details, refer to the software manual “http://www.compbio.med.harvard.edu/BIC-seq/.” For the segmentation step, parameters were designed as binsize = 50,000 bp and λ = 2 to determine the final CNV breakpoints.

### Construction of genome-wide chromatin interaction matrix using MGA-Seq datasets

FastQC (version: 0.11.5) was used to assess the quality of raw reads. FASTP (version: 0.23.2) was used to filter out the low-quality bases and adapter sequences. All the remaining read pairs were used to generate the chromatin contacts matrix file (.hic) using Juicer software (version: 1.6) [58]. For more details, refer to the software manual “https://github.com/aidenlab/juicer.”

### Identification of translocations types and breakpoints using MGA-Seq datasets

The chromatin contacts matrix file (.hic) was imported into Juicerbox (version: 1.9.8, https://github.com/aidenlab/Juicebox) software for visualization. The translocations and large structural variations were identified according to the inter-/intra- chromosome interaction patterns [7, 59]. The types and breakpoints of translocations were identified according to the split reads and CNV information. For unbalanced translocations, the chromosomal copy number at the breakpoint was usually altered, while balanced translocations do not have any chromosomal copy number changes.

### Identification of SNP and indel using in situ Hi-C datasets

The in situ Hi-C datasets of the SW480 cell line [38] were downloaded from Gene Expression Omnibus (GEO Accession: GSM3930294 and GSM3930295). The Hi-C ligation junction sequence “GATCGATC” and bases behind the ligation junction were removed by FASTP (version: 0.23.2). An example command line is as follows:

1. fastp -i insitu_sw480_1.fq -o trim_sw480_1.fq -w 15 –adapter_sequence GATCGATC
2. fastp -i insitu_sw480_2.fq -o trim_sw480_2.fq -w 15 –adapter_sequence GATCGATC

Trimmed reads1 and reads2 were merged together by the command line “cat trim_sw480_1.fq trim_sw480_2.fq > sw480_1_2.fq.” The merged reads file was used to identify SNPs and indels using the same parameters as MGA-Seq.

### Identification of CNV and translocation by in situ Hi-C datasets

All the raw Hi-C read pairs were used to detect CNVs. FastQC (version: 0.11.5) was used to assess the quality of raw reads, and FASTP (version: 0.23.2) was used to filter out the low-quality bases and adapter sequences. The CNV calling was carried out by BIC-seq2 [57]. The observed values were the residuals from GAM Poisson regression, and the expected values were set to zero. Translocation detection was performed by HINT-TL as implemented in HINT [33], a computational method for detecting CNVs and translocations based on Hi-C data.

### Identification of SNP, indel, and translocations using WGS datasets

FastQC (version: 0.11.5) was used to evaluate the quality of raw reads. FASTP (version: 0.23.2) was used to filter out the low-quality bases and adapter sequences. The trimmed reads pairs were used to identify SNPs and indels. The parameters are exactly the same as MGA-Seq.

Structural variation identification was carried out using Delly2 [60] (version: 0.8.6) and Gridss [61] (version: 2.12.2) with default parameters. One WGS data from a healthy person served as a control. Translocations that passed the internal quality control were merged with SURVIVOR [62] (version: 1.0.7, parameters: 1000 1 1 1 0 30). Only translocations supported by at least one definite split alignment read were retained.

### Genome-wide interaction fluctuation analysis (GWIFA)

According to the inter-chromosomal interaction feature of ecDNA and HSR, we designed a genome-wide interaction fluctuation analysis (GWIFA) to further characterize the inter-chromosomal interaction fluctuation of the focal amplification regions and defined a fluctuation score (FS) to distinguish ecDNA from HSR.

Firstly, we divided the genome into fixed-sized bins (100 kb) and calculated the cumulative interaction intensity (CII) between the focal amplified regions and the whole genome (Fig. 4j–m).

$${{CII}}_{x}= \sum_{i=1}^{x}{C}_{i}$$

In the formula, *x* represents the genome position measured by the number of bins, and *C*_*i*_ represents the number of contact counts inside the ith bin. We recommend a linear fit on CII, which can eliminate the abnormal fluctuations caused by uneven sequencing.

Next, second-order backward difference (SOBD) was introduced to further characterize the fluctuation of interactions across the genome (Fig. 4l, m). Denoting SOBD of CII as SOBDc.

$${{SOBD}c}_{x}=\frac{\left(\sum_{i=x-h+1}^{x}{C}_{i}-\sum_{j=x-2h+1}^{x-h}{C}_{j}\right)}{{h}^{2}}$$

In the formula, *h* is a customizable space, default is 3.

We then defined a fluctuation score (FS) to distinguish ecDNA from HSR.

$$\mathrm{FS} =\frac{\sum_{i=1}^\frac{n}{10}{S}_{i}}{\sum_{j=1}^{n}{S}_{j}}$$

In the formula, *S* is descending sorted distribution of |SOBDc| (absolute value of SOBDc), *n* is the quantity of SOBDc, and *T* is a customizable parameter (*T* < 1).

ecDNA and HSR can be distinguished as follows:

$$\mathrm{Discrimination}=\left\{\begin{array}{c}ecDNA, FS<T \\ HSR, FS>T\end{array}\right.$$

The complete analysis pipeline is available at https://github.com/yanyanzou0721/GWIFA.

### Long-read sequencing (Nanopore) data analysis

The nanopore sequencing reads with a quality score of more than 7 were mapped to the reference genome hg19 using minimap2 [63] (version: 2.17, -ax map-ont). Structural variants were called using NanoSV [64] (version: 1.2.4) with default parameters. Only SV supported by at least one definite split alignment read was retained for subsequent statistics.

### RNA-seq data analysis

FastQC (version: 0.11.5) was used to assess the quality of the raw reads. FASTP (version: 0.23.2) was used to filter out the low-quality bases and adapter sequences. The clean reads were aligned to the hg19 using BWA-MEM (version: 0.7.17) with default parameters and sorted by Samtools (version: 1.15.1). Gene expression levels were assessed using featureCounts [65] (version: 2.0.0). Differential gene expression analysis was performed using DEseq2 [66] (version: 1.20.0) package.

### Supplementary Information

**Additional file 1: Fig. S1-S9.** Supplementary figures.

**Additional file 2: Table S1.** The split reads located at the boundaries of focally amplified regions in K562 cells.

**Additional file 3: Table S2.** The regions of focal amplification in the renal cancer tissue.

**Additional file 4.** Review history.

## Data Availability

All sequencing data generated in this study have been deposited in the Gene Expression Omnibus (GEO) under accession GSE205293 [67]. The Hi-C datasets of SW480 cell line were downloaded from GEO (Accession GSM3930294 [68] and GSM3930295 [69]). GWIFA is available at GitHub [70] and Zenodo [71].
