# Supplementary material for: MGA-seq: robust identification of extrachromosomal DNA and genetic variants using multiple genetic abnormality sequencing
Source: Genome Biol. 2023 Oct 30;24:247. doi: 10.1186/s13059-023-03081-x (PMC10614391; doi:10.1186/s13059-023-03081-x)
Supplement: Supplementary file 1 — Additional file 1: Fig. S1-S9. Supplementary figures. [file 13059_2023_3081_MOESM1_ESM.docx]

**Supplementary Figures**


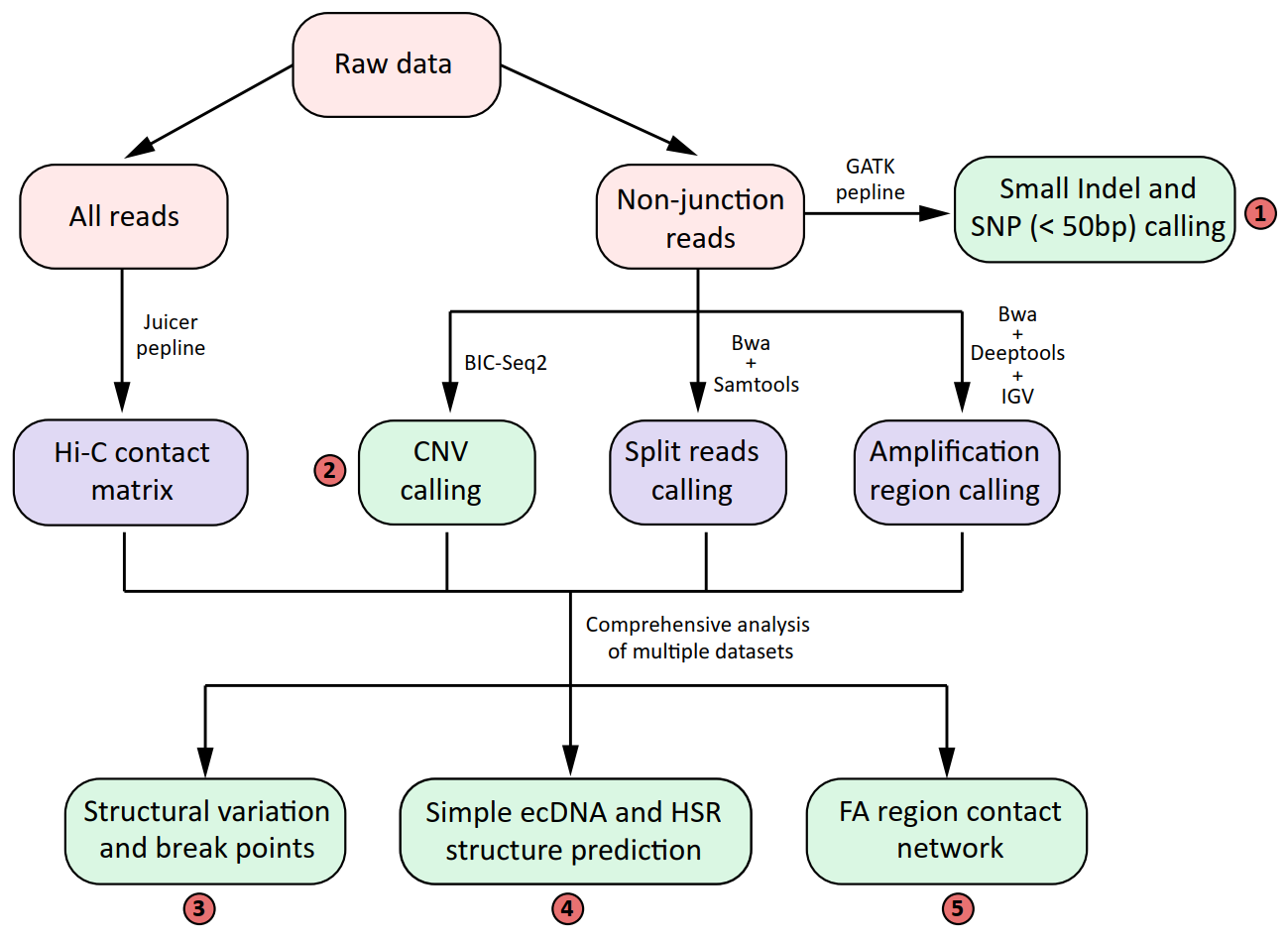


**Fig. S1: Flow-chart of MGA-Seq data analysis.** After sequencing, all sequencing reads were used to generate chromatin contact matrix by juicer pipeline. The reads without proximity ligation junction were used to detect small indels and SNPs (< 50bp), CNVs, split reads, and genomic amplification regions. With the integrated analysis of chromatin contact matrix, these datasets can be used to decode the type and breakpoints of translocations, distinguish ecDNA from HSR, predict the focal amplification structure, and construct FA region interaction network.


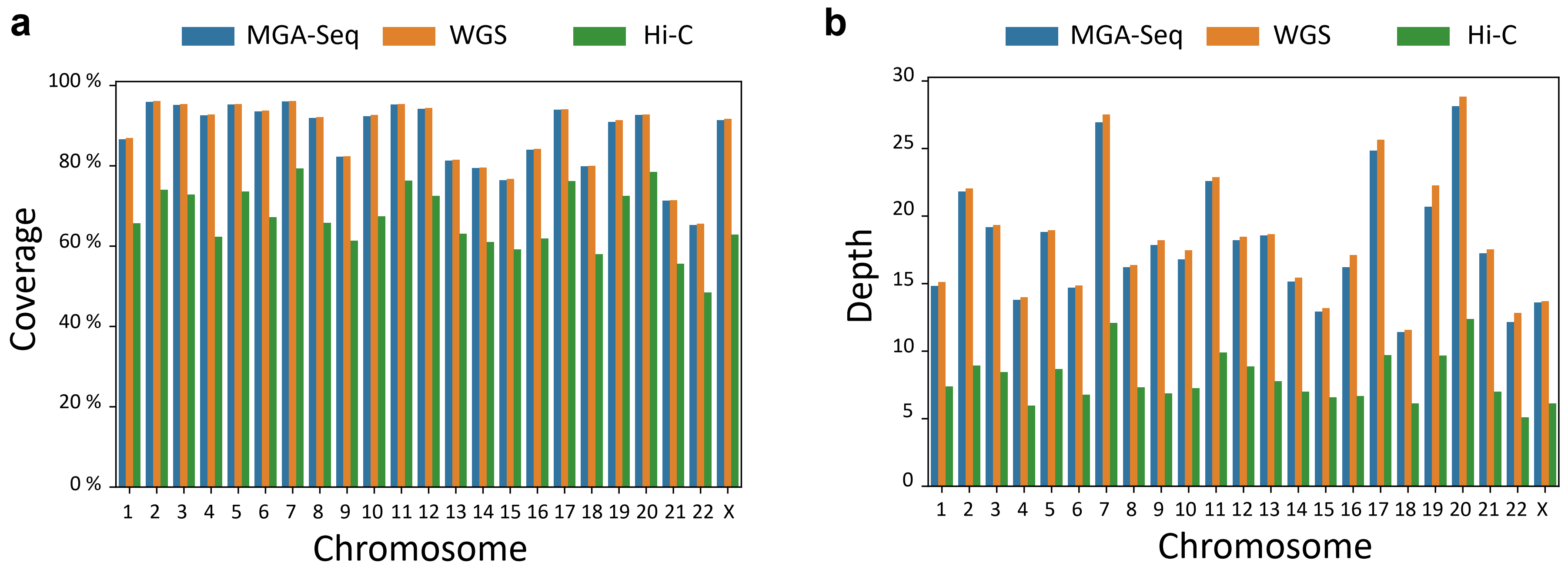


**Fig. S2: Comparison of the sequencing coverage and depth of MGA-Seq, WGS, and Hi-C in the SW480 cell line. a,** Histogram of coverage for each chromosome. Blue represents MGA-Seq (raw reads: 189,873,250; PE150), yellow represents WGS (raw reads: 194,167,430; PE150), and green represents Hi-C (raw reads: 299,462,905; PE75). **b,** Histogram of sequencing depth for each chromosome. Blue represents MGA-Seq (raw reads: 189,873,250; PE150), yellow represents WGS (raw reads: 194,167,430; PE150), and green represents Hi-C (raw reads: 299,462,905; PE75).


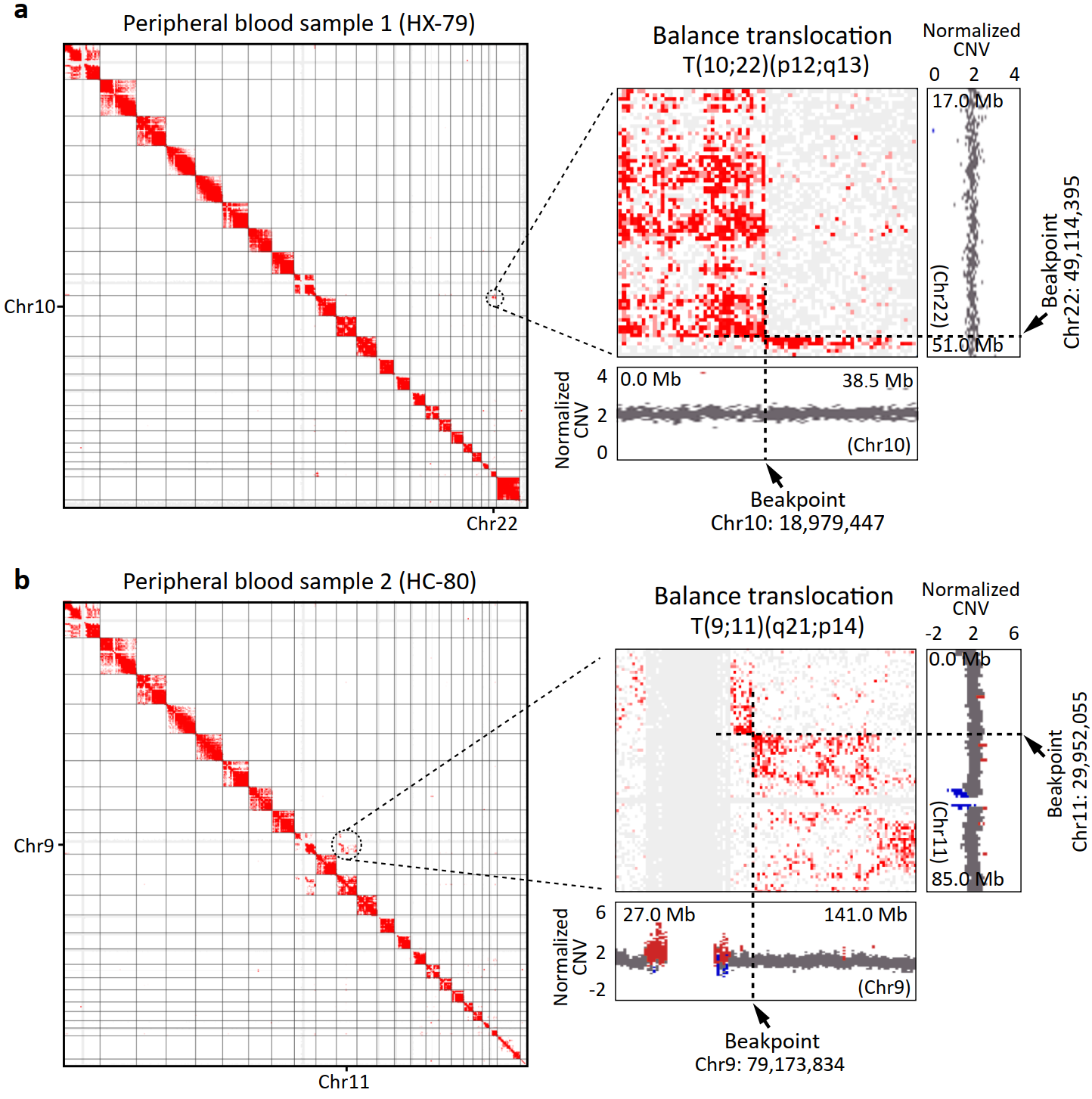


**Fig. S3: Identification of translocation types and breakpoints by MGA-Seq. a,** Identification of balance translocation T(10;22)(p12;q13) and genome breakpoint in patient 1. **b,** Identification of balance translocation T(9;11)(q21;p14) and genome breakpoint in patient 2.


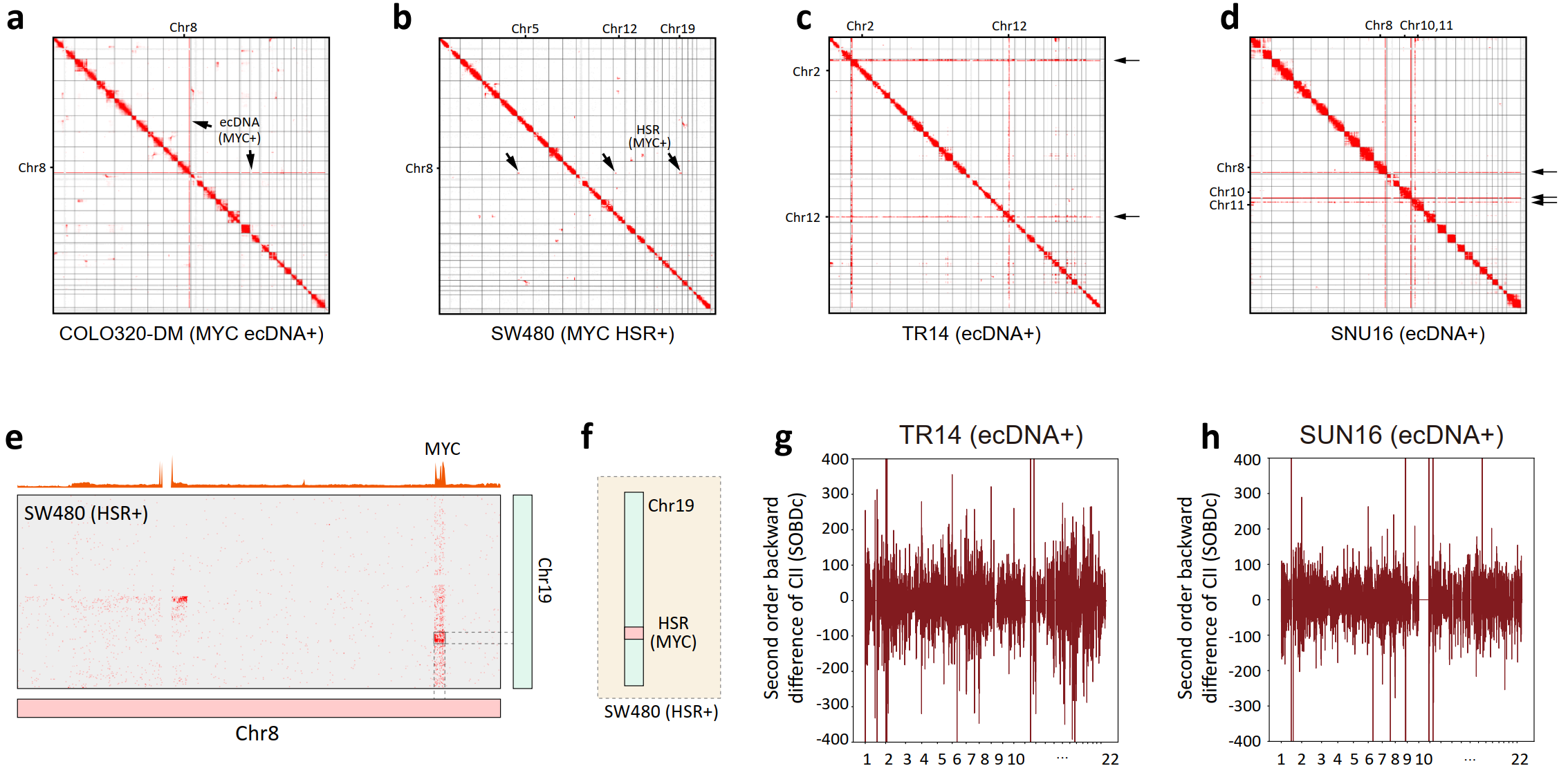


**Fig. S4: Chromatin contact matrix and genome-wide interaction fluctuation analysis (GWIFA) of ecDNA-positive cell lines. a-d,** Genome-wide chromatin contact matrix of COLO320-DM, SW480, TR14, and SUN16 cell lines. The amplified regions are marked with arrows. **e,** The chromatin interaction matrix of SW480 cell line between chr 8 and chr 19. The *MYC* amplified region is marked with a dashed line in the figure. **f,** MYC is amplified in the form of HSR on chr 19. **g and h.** Plotted the second-order backward difference (SOBD) values across the genome for the focal amplification region of TR14 (chr12:69,000,000-70,000,000) and SUN16 (chr10:122,100,000-123,600,000) using a bin size of 100 kb.


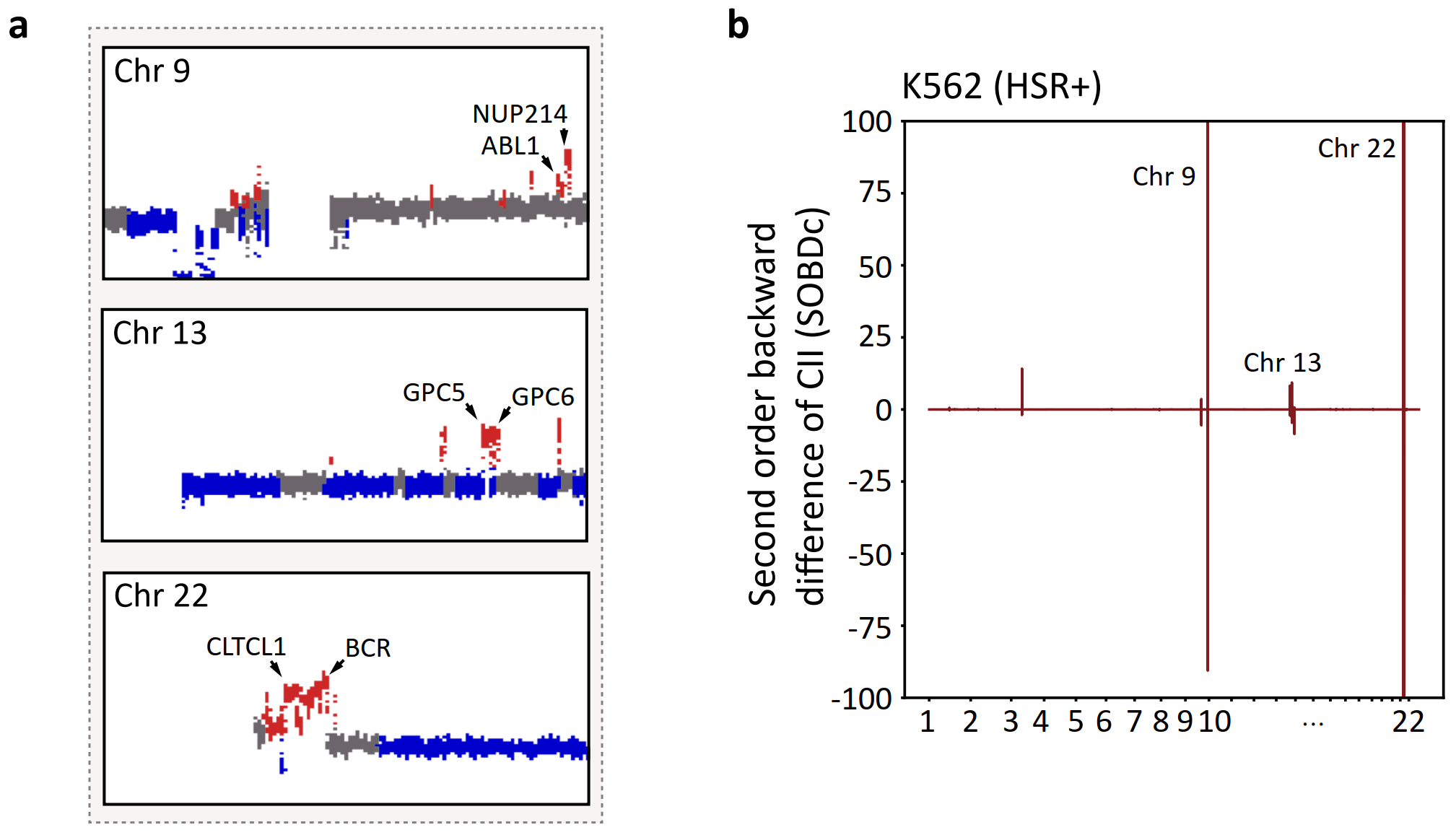


**Fig. S5: Copy number variation (CNV) analysis of K562 cell line. a,** CNV analysis of chromosomes 9, 13, and 22 in K562 cell line. Gains and losses of copy number are shown in red and blue, respectively. Representative genes located in amplification region are marked with arrows. **b,** Plotted the second-order backward difference (SOBD) values across the genome for the focal amplification region of K562 (chr22:18,827,319-23,707,746) using a bin size of 100 kb.


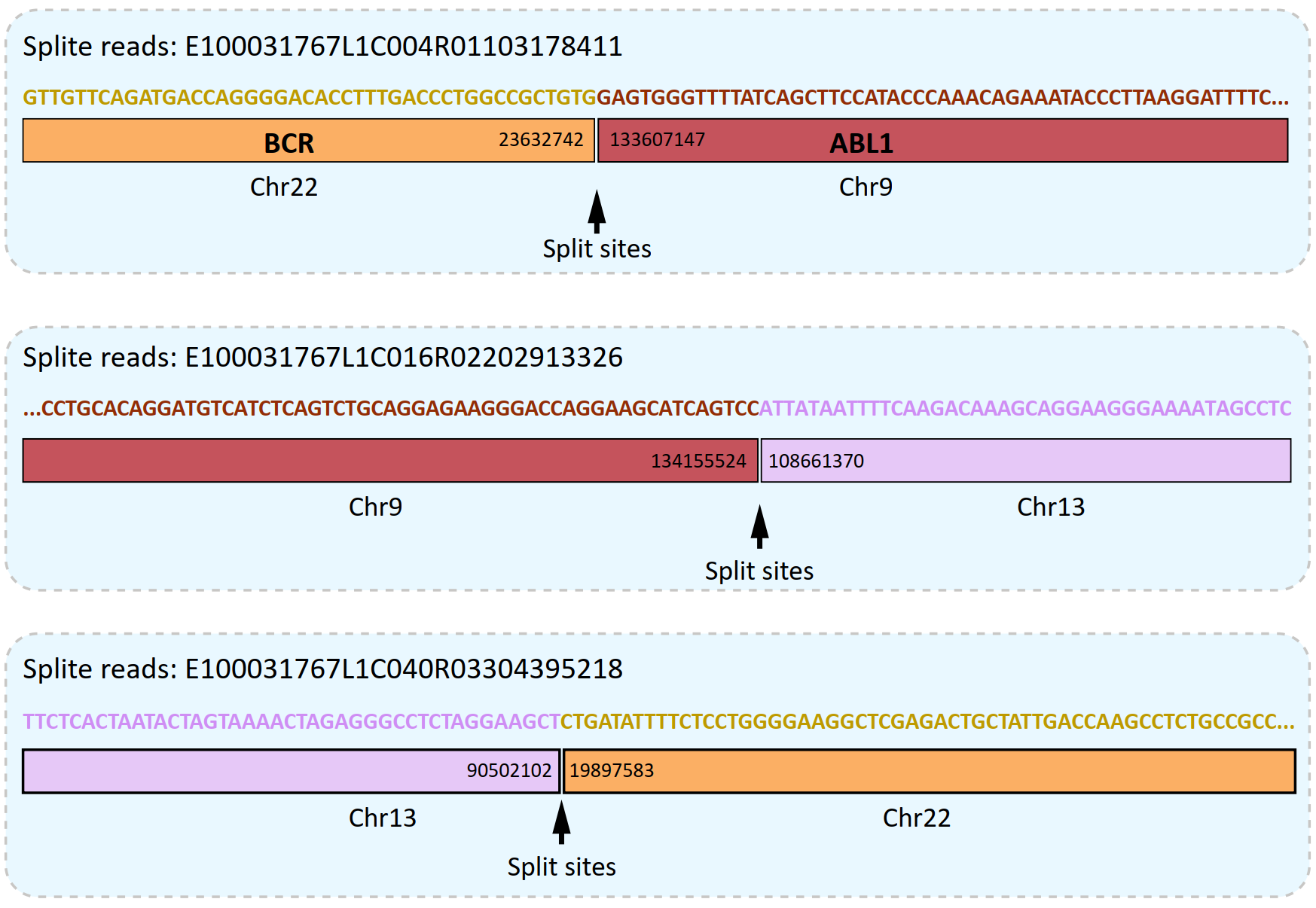


**Fig. S6: Sequence and breakpoints of split reads used to assemble HSR in K562 cells.**


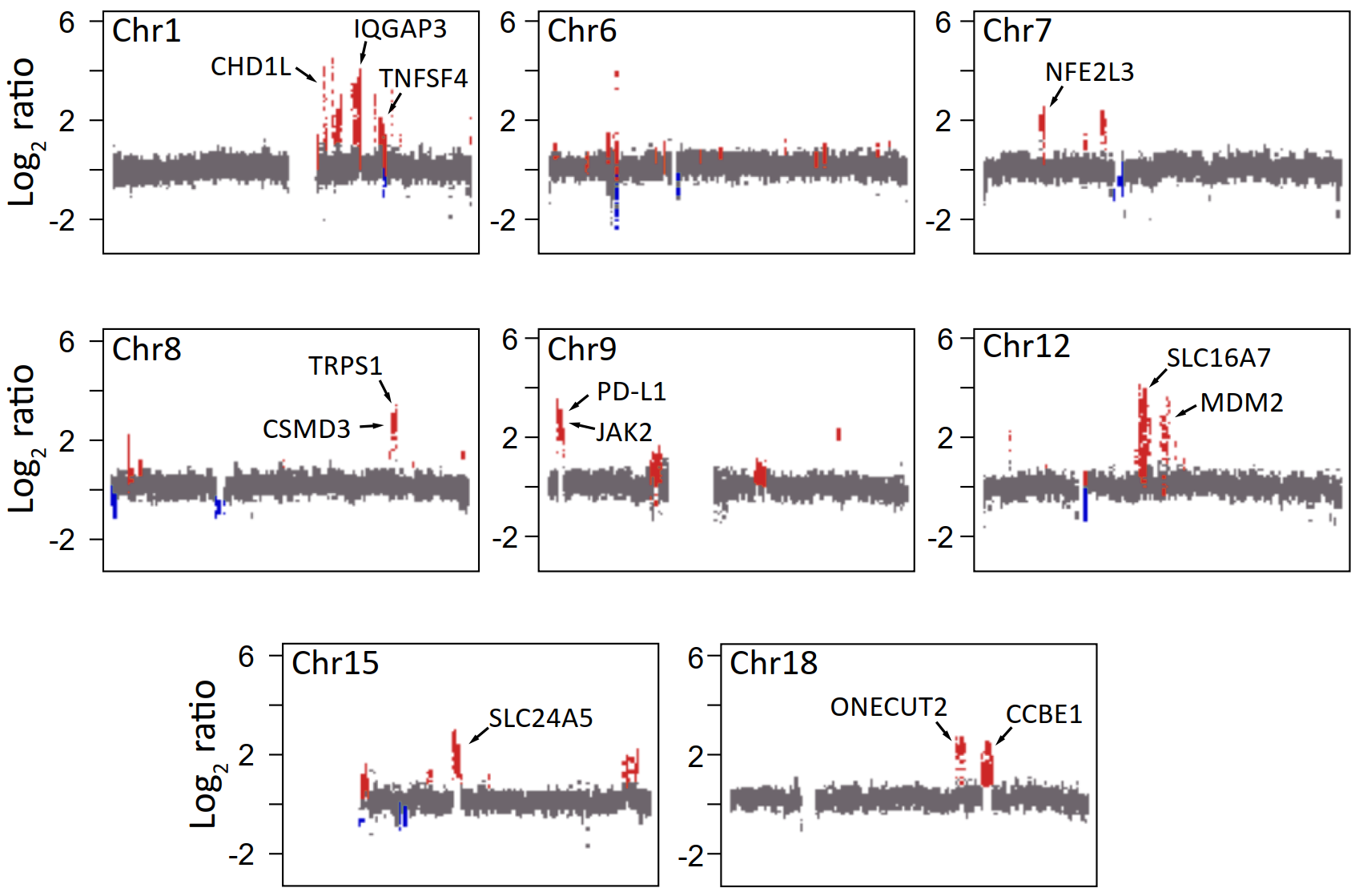


**Fig. S7: CNV analysis of renal cancer tissue.** CNV analysis of chromosomes with abnormal amplification in renal cancer tissue. Gains and losses of copy number are shown in red and blue, respectively. Representative genes located in amplification region are marked with arrows.


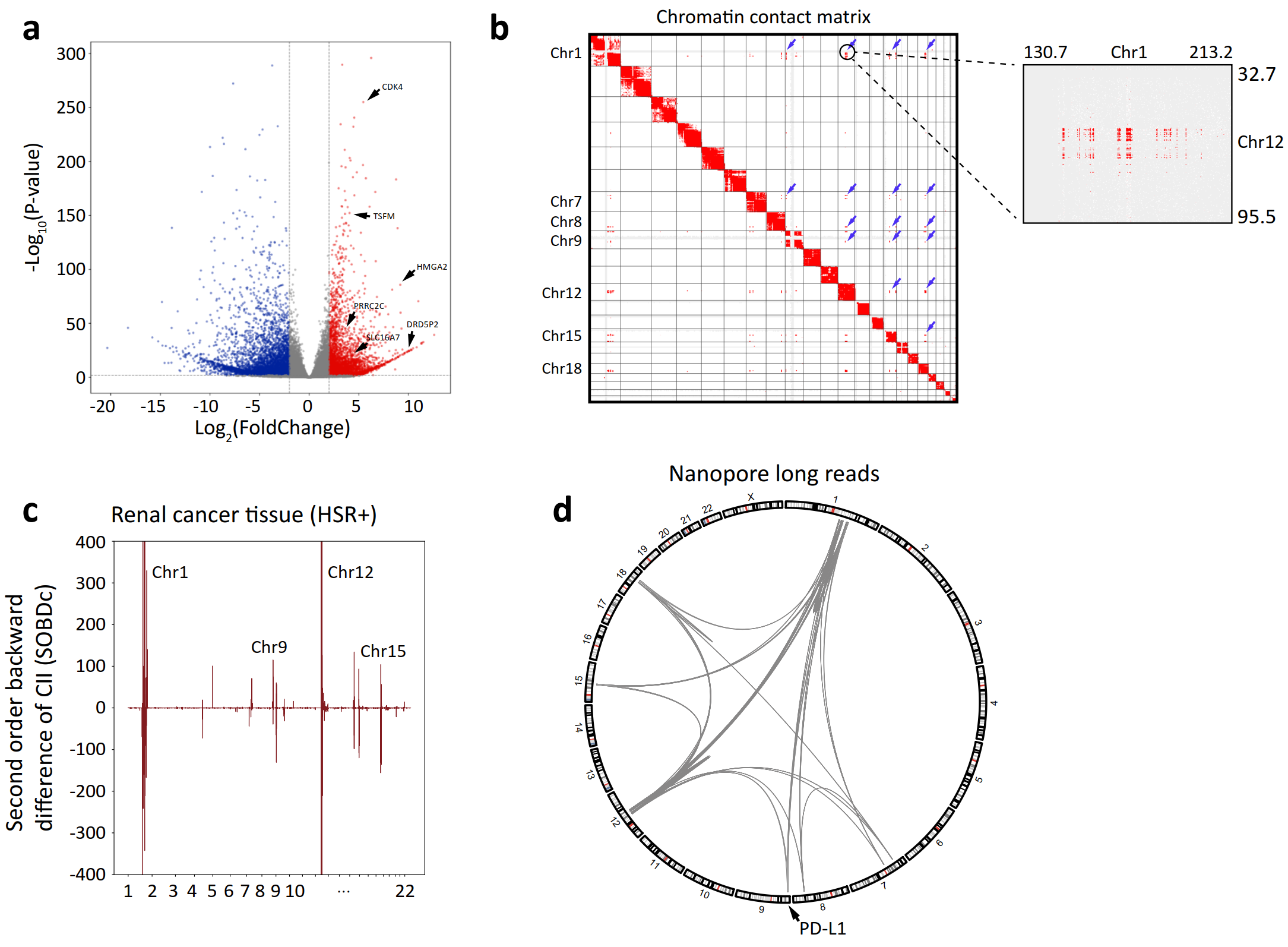


**Fig. S8: Verification of inter and intra chromosomal interaction between focal amplification regions in renal cancer tissue by nanopore. a,** Volcano plots of differential expression genes between renal cancer tissue and normal kidney tissue control. **b,** Genome-wide chromatin contact matrix of renal cancer tissue. Potential HSR regions are marked with arrows in the figure. The inter-chromosomal contacts between the focal amplification regions and Chr1 and Chr12 are zoomed in. **c,** Plotted the second-order backward difference (SOBD) values across the genome for the focal amplification region (chr12:58,000,000-68,000,000) of the renal cancer tissue using a bin size of 100 kb. **d,** Validation of split reads and chromatin interactions across focal amplification regions with Nanopore long reads.


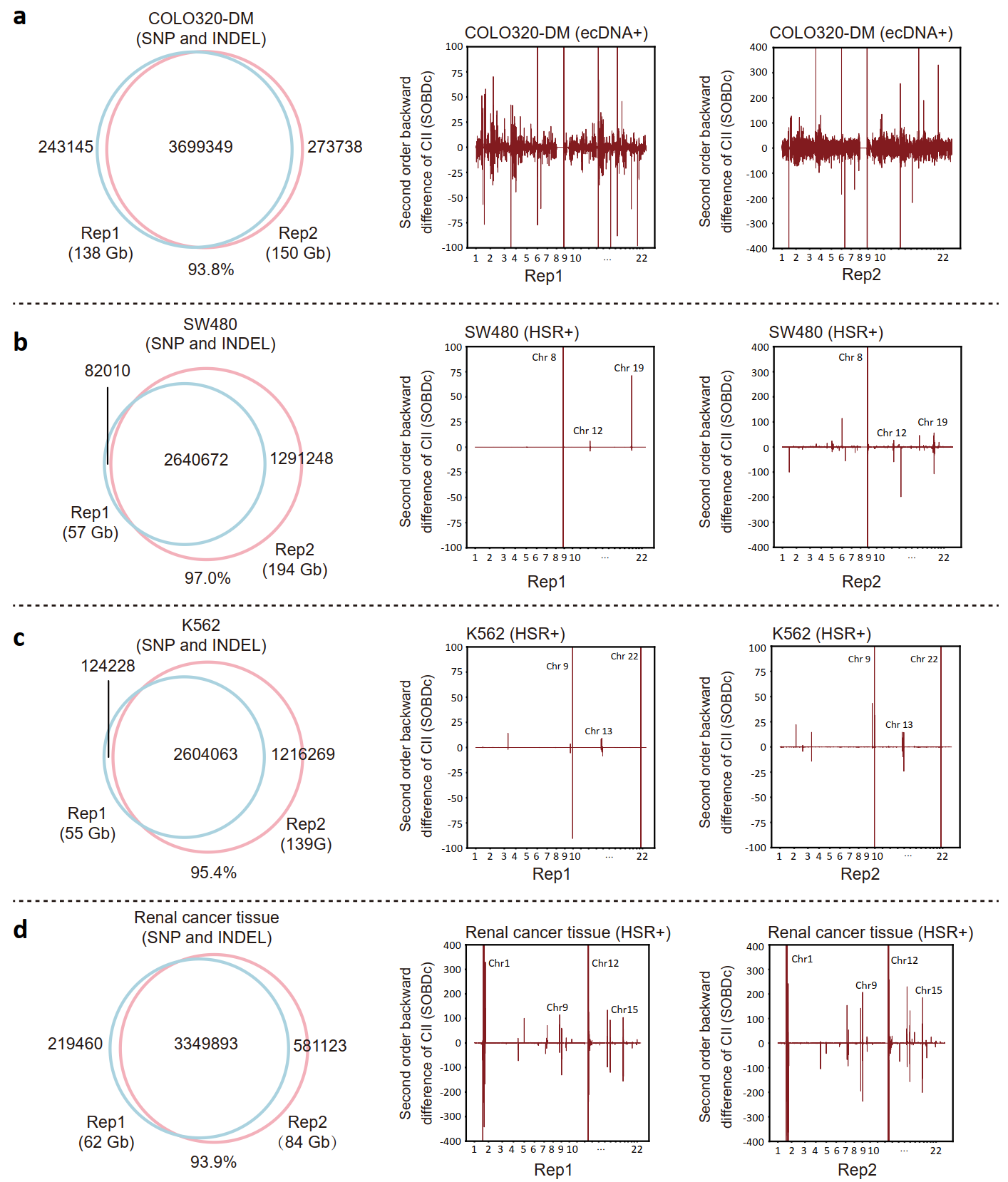


**Fig. S9: Consistency of SNP, INDEL, and FA type identification in two replicate experiments: a-d,** Two independent replicate experiments were conducted on COLO320-DM, SW480, K562, and renal cancer tissue samples for SNP, INDEL, and FA type identification. In replicate experiment one (rep1), 93.8%-97.0% of SNPs and INDELs were successfully detected again in replicate experiment two (rep2). Furthermore, the types of Focal Amplifications (FAs) identified in both replicate experiments were found to be completely consistent.
