# Supplementary material for: MGA-seq: robust identification of extrachromosomal DNA and genetic variants using multiple genetic abnormality sequencing
Source: Genome Biol. 2023 Oct 30;24:247. doi: 10.1186/s13059-023-03081-x (PMC10614391; doi:10.1186/s13059-023-03081-x)
Supplement: Supplementary file 4 — Additional file 4. Review history. [file 13059_2023_3081_MOESM4_ESM.docx]

**1^st^ round**

**Reviewer 1**

The authors developed a protocal that can detect a multitude of genetic abnormality of cancer.
Their procedure is suitable and efficient and this reviewer recommands the manuscript to be published.

This kind of one stop diagnostic method for cancer using the structural variation information from DNA sources is an important technology.
The technique deserves publication for its simplicity and low-cost (author's claim. ~60 USD per preparation).

1. Authors should add the actual protocal in the abstract. (It is currently just a claim summary).
"approximate location of genomic structural variation, it can facilitate breakpoint searching." is a description on what MGA-seq is and this kind of information should be included in abstract.

2. Please put the comparison points on the difference between MGA-seq and Hi-C method in the background.
3. The authors repeat their results in Discussion. They should remove those and put information/discussion on the limitation of MGA-seq.

**Reviewer 2**

The authors present a new sequencing method MGA-Seq, which combines low-mid coverage WGS with aspects of Hi-C, to which they compare the results. The new method appears to give similar performance to similar depth WGS, and is better than Hi-C when measuring copy number, SNPs etc, which is not surprising. They demonstrate utility in identifying focal amplifications and ecDNA.

The main weakness of the study is the lack of data on reproducibility and robustness of the method. Every experiment appears to have been done just the once. The evaluation of performance would be improved by knowing in particular whether the ecDNA interactions are reproducible and whether the same SNPs are detected each time. Indeed it would be interesting to know how reproducible eCDNA interactions are if measured at a different time point. Are they associating with the same regions reproducibly, or do they move around?

When compared to WGS only 83% overlap of SNPs and this is in cell lines - what would it be like in tumours with potentially contaminating normal DNA? Fig S2 shows a coverage 15-30x for MGAseq and WGS (almost identical). Are those that are missing in areas of low coverage/depth?

The cost is given as $56 but this does not include sequencing, which presumably would be similar to WGS of same depth.

Line 176 "190 million raw reads, the structural variants detected by MGA-Seq were completely consistent with in situ Hi-C with 300 million raw reads (Fig. 3a)."
However the read lengths were 150bp vs 75bp so this isn't the best comparison. Perhaps present the average coverage instead.

Fig 2e - what is log2 scale of?

Fig 4h,I and 5a, 6a what is Y axis? Units missing on 6a RNAseq

Line 363 - something missing?

Fig S2 - is this all samples or a particular sample (specify)? If all samples, can you show each? This is actually an important figure - I would have liked to see this presented with as part of Fig 2, even if only panel "a".

**Authors’ response**

We’d like to thank all the reviewers for their critical comments, inspiring suggestions and kind considerations. We performed substantial experiments according to the reviewers’ comments and suggestions, which have further strengthened our study.

Reviewer #1:

The authors developed a protocal that can detect a multitude of genetic abnormality of cancer. Their procedure is suitable and efficient and this reviewer recommands the manuscript to be published.

This kind of one stop diagnostic method for cancer using the structural variation information from DNA sources is an important technology.

The technique deserves publication for its simplicity and low-cost (author's claim. ~60 USD per preparation).

1. Authors should add the actual protocal in the abstract. (It is currently just a claim summary). "approximate location of genomic structural variation, it can facilitate breakpoint searching." is a description on what MGA-seq is and this kind of information should be included in abstract.

Response: We appreciate the important suggestions and comments. As suggested, we have modified the abstract and incorporated a description of the MGA-Seq method, which can be found between lines 57 and 60.

2. Please put the comparison points on the difference between MGA-seq and Hi-C method in the background.

Response: Thanks for the suggestion. We have added a detailed comparison of MGA- Seq and Hi-C in the revised background section (lines 121 and 140). In this section, we pointed out the limitations of the Hi-C method when it comes to detecting small-scale mutations and deletions, and elaborate on how to improve these issues by MGA-Seq.

3. The authors repeat their results in Discussion. They should remove those and put information/discussion on the limitation of MGA-seq.

Response: We are grateful for the reviewer's suggestion. As suggested, we have revised the Discussion section to avoid repetition with the Results section. Also, we have discussed the limitations of MGA-Seq from lines 384 to 391.

Reviewer #2:

The authors present a new sequencing method MGA-Seq, which combines low-mid coverage WGS with aspects of Hi-C, to which they compare the results. The new method appears to give similar performance to similar depth WGS, and is better than Hi-C when measuring copy number, SNPs etc, which is not surprising. They demonstrate utility in identifying focal amplifications and ecDNA.

The main weakness of the study is the lack of data on reproducibility and robustness of the method. Every experiment appears to have been done just the once. The evaluation of performance would be improved by knowing in particular whether the ecDNA interactions are reproducible and whether the same SNPs are detected each time.

Response: We appreciate the reviewer's suggestions and comments. To demonstrate the reproducibility of our MGA-Seq method, we reconstructed the MGA-Seq libraries of COLO320-DM, SW480, K562, as well as the renal cancer tissue used in Fig 6 for sequencing. As shown in Fig R1. A-D, there is a high correlation (93.8%-97.0%) between both replicates. Furthermore, we have validated the types of amplification through genome-wide interaction fluctuation analysis (GWIFA) again by another replicate, which yield consist result with our previous data (Fig R1: A-D). These results underscore the reliability and reproducibility of our MGA-Seq method.

Fig R1. Consistency of SNP, INDEL, and FA detection in two replicate experiments: a-d, Two independent replicate experiments of COLO320-DM, SW480, K562, and renal cancer tissue samples for SNP, INDEL, and FA detection. In replicate experiment one (rep1), 93.8%- 97.0% of SNPs and INDELs were successfully detected again in replicate experiment two (rep2). Furthermore, the types of Focal Amplifications (FAs) identified in both replicate experiments were consistent.

Indeed it would be interesting to know how reproducible ecDNA interactions are if measured at a different time point. Are they associating with the same regions reproducibly, or do they move around?

Response: Thanks for this important question. To test whether the interactions of ecDNA are reproducible at different time points, we collected COLO320-DM cells after 24 and 48 hours of culture, respectively, and analyzed the interactions between ecDNA amplification regions and chromosomes Fig R2: A, B. As shown in Fig R2: C, D, 38.3% of the interactions at 24 hours are consistent with the interactions observed at 48 hours.

When compared to WGS only 83% overlap of SNPs and this is in cell lines - what would it be like in tumours with potentially contaminating normal DNA?

Response: We greatly appreciate this crucial question. In the SW480 sample, the overlap rate of SNPs and INDELs between MGA-Seq and WGS is only 83%, which is most likely due to sequencing depth. When we increase the sequencing depth of MGA- Seq to approximately ~60x (194GB), 97.1% of the SNPs and INDELs identified by WGS can be re-identified by MGA-Seq (Fig R3. A). In Fig 6 of our manuscript, the renal cancer tissue indeed contains a certain degree of normal adipose cells, as shown in the HE staining results in Fig R3.B. To validate the consistency of our MGA-Seq method in identifying SNPs and INDELs in this tissue samples with WGS, we performed WGS sequencing on this renal cancer tissue sample. Our data showed that 97.6% of the SNPs and INDELs could be detected again by WGS, demonstrating the robustness of MGA-Seq in mutation detection in tissue samples with normal DNA (Fig R3. C).

Fig R3. Validation of MGA-Seq's capability in detecting SNPs and INDELs in tumor tissue samples. A. Comparison of MGA-Seq's ability to detect SNPs and INDELs in the SW480 cell line at different sequencing depths. B. Hematoxylin and eosin (HE) stained image of renal cancer tissue samples, with normal cells indicated by dashed lines. C. Comparison of MGA- Seq's consistency with Whole Genome Sequencing (WGS) in detecting SNPs and INDELs in tissue samples.

Fig S2 shows a coverage 15-30x for MGA-seq and WGS (almost identical). Are those that are missing in areas of low coverage/depth?

Response: We appreciate the reviewer's question. In MGA-Seq and WGS data, the lower coverage of certain chromosomes may not be due to sequencing depth alone. In some chromosomes, regions such as telomeres, centromeres, and the short arm areas contain a large number of repetitive sequences. These repetitions lead to a lower mapping rate of the sequencing reads, thereby cause some missing areas in these specific chromosomes (Fig R4. A, highlighted with red arrows). In line with this, even we increased the sequencing depth from ~18x (57 Gb) to ~61x (194 Gb) for the MGA- Seq data of SW480, the coverage of chromosomes in chr13, chr14, chr15, chr21, and chr22 does not show a significant improvement (Fig R4. B)

Fig R4. Comparison of genome coverage with different sequencing depths using MGA- Seq. A. Genome coverage and sequencing depth of MGA-Seq (57 Gb raw reads). Chromosomes with abundant repetitive sequences in the short arm regions are indicated by red arrows. B. Comparison of genome coverage of different chromosomes with MGA-Seq at sequencing data volumes of 57 Gb and 194 Gb.

The cost is given as $56 but this does not include sequencing, which presumably would be similar to WGS of same depth.

Response: The current sequencing cost on the Illumina X Plus platform is approximately $4 per Gb. With a sequencing depth of 60 Gb per sample, the total cost of MGA-Seq is $56 + $240 = $296, and the total cost of WGS is $30 + $240 = $270.

Line 176 "190 million raw reads, the structural variants detected by MGA-Seq were completely consistent with in situ Hi-C with 300 million raw reads (Fig. 3a)."

However the read lengths were 150bp vs 75bp so this isn't the best comparison. Perhaps present the average coverage instead.

Response: We greatly appreciate the reviewer's valuable comment. As suggest, we have presented the average coverage data into the revised Fig. 3a.

Fig 2e - what is log2 scale of?

Response: We apologize for the oversight. Here, Log2 refers to taking the logarithm (base 2) of the CNV numbers. We have clarified this in the figure.

Fig 4h,I and 5a, 6a what is Y axis? Units missing on 6a RNAseq

Response: We apologie for the lack of clarity in our previous explanation. The Y-axis in Figures 4h, 4i, 5a, and 6a represents the reads that were normalized based on the total number of reads mapped per sample. We have clarified this in the revised figure legends. In the RNA-Seq subplot of Figure 6a, we have added the units.

Line 363 - something missing?

Response: Thank you very much for pointing out the oversight. We have made corrections in the revised discussion section.

Fig S2 – is this all samples or a particular sample (specify)? If all samples, can you show each? This is actually an important figure – I would have liked to see this presented with as part of Fig 2, even if only panel “a”.

Response: In Fig. S2, we had calculated for a specific sample, which is the same sample analyzed in Fig. 2a. We greatly appreciate the suggestion from the reviewer and have now moved Fig. S2a to Fig. 2c.

**2^nd^ round**

**Reviewer 1**

The revision is good and this new ma-seq can be useful for genomics applications.
I recommend it to be publicized asap.
